# Validation study of genetic biomarkers of response to TNF inhibitors in rheumatoid arthritis

**DOI:** 10.1371/journal.pone.0196793

**Published:** 2018-05-07

**Authors:** Rosario Lopez-Rodriguez, Eva Perez-Pampin, Ana Marquez, Francisco J. Blanco, Beatriz Joven, Patricia Carreira, Miguel Angel Ferrer, Rafael Caliz, Lara Valor, Javier Narvaez, Juan D. Cañete, Maria del Carmen Ordoñez, Sara Manrique-Arija, Yiannis Vasilopoulos, Alejandro Balsa, Dora Pascual-Salcedo, Manuel J. Moreno-Ramos, Juan Jose Alegre-Sancho, Federico Navarro-Sarabia, Virginia Moreira, Rosa Garcia-Portales, Enrique Raya, Cesar Magro-Checa, Javier Martin, Juan J. Gomez-Reino, Antonio Gonzalez

**Affiliations:** 1 Experimental and Observational Rheumatology and Rheumatology Unit, Instituto de Investigación Sanitaria, Hospital Clínico Universitario de Santiago, Santiago de Compostela, Spain; 2 Instituto de Parasitología y Biomedicina López-Neyra, CSIC, Granada, Spain; 3 Rheumatology Department, Instituto de Investigacion Biomedica–Complejo Hospitalario Universitario A Coruna, Coruna, Spain; 4 Reumatology Department, Madrid, Spain; 5 Rheumatology Unit, Hospital Universitario Virgen de las Nieves, Granada, Spain; 6 Rheumatology Unit, Hospital General Universitario Gregorio Marañón, Madrid, Spain; 7 Department of Rheumatology, Hospital Universitario de Bellvitge, Barcelona, Spain; 8 Arthritis Unit, Rheumatology Dpt, Hospital Clinic and IDIBAPS, Barcelona, Spain; 9 Servicio de Reumatología, HRU Carlos Haya, Universidad de Málaga, Instituto de Investigación Biomédica de Málaga (IBIMA), Málaga Spain; 10 Department of Biochemistry and Biotechnology, University of Thessaly, Larissa, Greece; 11 Rheumatology Unit, Instituto de Investigación Sanitaria del Hospital Universitario La Paz (IdiPAZ), Hospital Universitario La Paz, Madrid, Spain; 12 Department of Immunology, Instituto de Investigación Hospital Universitario La Paz (IdiPAZ), Madrid, Spain; 13 Department of Rheumatology, Hospital Virgen de la Arrixaca, Murcia, Spain; 14 Department of Rheumatology, Hospital Doctor Peset, Valencia, Spain; 15 Rheumatology Unit, Hospital Universitario Virgen Macarena, Sevilla, Spain; 16 Department of Rheumatology, Hospital Virgen de la Victoria, Málaga, Spain; 17 Department of Rheumatology, Hospital Clínico San Cecilio, Granada, Spain; 18 Department of Rheumatology, Leiden University Medical Center, Leiden, The Netherlands; University of Arkansas for Medical Sciences, UNITED STATES

## Abstract

Genetic biomarkers are sought to personalize treatment of patients with rheumatoid arthritis (RA), given their variable response to TNF inhibitors (TNFi). However, no genetic biomaker is yet sufficiently validated. Here, we report a validation study of 18 previously reported genetic biomarkers, including 11 from GWAS of response to TNFi. The validation was attempted in 581 patients with RA that had not been treated with biologic antirheumatic drugs previously. Their response to TNFi was evaluated at 3, 6 and 12 months in two ways: change in the DAS28 measure of disease activity, and according to the EULAR criteria for response to antirheumatic drugs. Association of these parameters with the genotypes, obtained by PCR amplification followed by single-base extension, was tested with regression analysis. These analyses were adjusted for baseline DAS28, sex, and the specific TNFi. However, none of the proposed biomarkers was validated, as none showed association with response to TNFi in our study, even at the time of assessment and with the outcome that showed the most significant result in previous studies. These negative results are notable because this was the first independent validation study for 12 of the biomarkers, and because they indicate that prudence is needed in the interpretation of the proposed biomarkers of response to TNFi even when they are supported by very low p values. The results also emphasize the requirement of independent replication for validation, and the need to search protocols that could increase reproducibility of the biomarkers of response to TNFi.

## Introduction

Rheumatoid arthritis (RA) is a systemic autoimmune disease mainly characterized by inflammation of synovial joints [1]. If poorly treated, RA is very painful and incapacitating, and it can lead to joint deformities, loss of job and mobility, and premature death. Currently, the prognosis is much better than before the turn of the XXI century thanks to drugs that are specifically targeted to immune mediators [1,2]. The first drugs of this class were the TNF inhibitors (TNFi), infliximab, and adalimumab, which are monoclonal anti-TNF antibodies, and etanercept, which is a recombinant soluble TNF receptor. Now, more TNFi are available together with other drugs targeting IL6, B cells, T cells or intracellular kinases. This range of drugs is welcomed because none of them is effective in all patients. Typically about 30% of the patients fail to respond to any of the drugs, and an additional 30% of patients show only a partial response. This between-patient variability has not prevented the rheumatologists to aim for remission or low disease activity, which they seek by changing from one drug to another, and by combining them with conventional antirheumatic drugs [1,2]. The election of drug follows a trial and error approach, which is very unsatisfactory because these drugs are expensive and have potential side effects. Furthermore, delayed control of the disease worsens long term prognosis. A very attractive alternative will be to use biomarkers for personalizing the treatment [3,4].

An important effort has been directed to the discovery of genetic biomarkers of response to TNFi [5,6]. It has involved candidate gene studies and Genome-Wide Association studies (GWAs). This effort has led to some remarkable findings: the two SNPs (rs3794271 and rs284511) that have achieved association with response to TNFi below the GWAS significance threshold of p < 5 x10^-8^ [7,8]; the association of the *PTPRC* locus in three large independent studies [9–11]; and two other SNPs (rs6427528 and rs113878252) with very convincing evidence of association with response to etanercept [12,13]. Regrettably, none of these results or any of the other proposed biomarkers is sufficiently validated, either because an independent validation is still pending, or because of lack of replication in other studies.

Here, our aim has been to validate 18 SNPs that were previously associated with response to TNFi in RA [7,8,14–18]. This list of SNPs includes some of the most promising genetic biomarkers mentioned above [7,8], and others [14–18]. Unfortunately, none of the SNPs showed association with response to TNFi in our RA patients.

## Materials and methods

### Patients

The study population consisted of 581 patients with RA according to the ACR 1987 criteria [19]. They were treated with an TNFi as the first targeted antirheumatic drug by indication of the attending rheumatologist. This treatment indication was made with independence of the study. Recruitment of samples was approved by the local ethics committees and patients gave their written informed consent. The study was conducted according to the principles of the Declaration of Helsinki (2013) and was approved by the Research Ethics Committee of Galicia (Spain, code 2014/387). The patients were of European origin (Spanish ancestry = 530 and Greek ancestry = 51). Change in Disease Activity Score 28 joints (ΔDAS28 = DAS28baseline − DAS28follow-up) was used as the primary outcome. The DAS28 composite index includes erythrocyte sedimentation rate, self-reported global patient health, and counts of swollen joints and of tender joints among 28 selected joints [20]. A score over 5.1 indicates high disease activity, whereas below 3.2 indicates low activity. In addition, response according to the European League Against Rheumatism (EULAR) criteria was assessed [21]. Non-responders are defined as patients showing ≤ 0.6 improvement from baseline DAS28, or showing modest improvement, ≤ 1.2, but remaining with high disease activity, DAS28 > 5.1. Good responders show DAS28 ≤ 3.2 and improvement from baseline > 1.2. The remaining patients are considered moderate responders.

### Genotyping

Eighteen SNPs were selected because of previous association with response to TNFi in RA irrespective of ethnicity or country of origin (Table A in S1 Text) [7,8,14–18]. Genotyping was conducted by multiplex PCR amplification followed by single-base extension with the SNaPshot Multiplex Kit (Applied Biosystems, Foster City, California). The primers, probes and detailed protocols used for these analyses are available from the authors upon request. Replication in duplicate genotyping reactions of 10% of the samples, genotype call rate ≥ 94%, concordance with the Hardy–Weinberg equilibrium (p<0.01), and with SNP frequencies in the HapMap collections [22], were used for quality control (Table B in S1 Text). Two SNPs (rs10520789 and rs1679568) did not follow the Hardy–Weinberg equilibrium and were excluded. The R statistical package SNPAssoc was used for Hardy-Weinberg equilibrium assessment and allelic frequency calculation [23].

### Statistical analysis

The main outcome of treatment was ΔDAS28 at 6 months. EULAR non-responders compared with responders (good + moderate) or non-responders compared with good responders (moderate responders left out) at any time, and ΔDAS28 at 3 or 12 months were considered secondary outcomes. Linear and logistic regression models for ΔDAS28 and EULAR response were fitted, respectively. Genotypes were considered in accordance with an additive genetic model of minor allele counts (0, 1 or 2). Therefore, positive regression coefficients indicate a better response associated with minor allele additive effects. Analyses were adjusted for baseline DAS28, sex and the specific TNFi. Results were interpreted considering the number of tests following the Bonferroni approach, consistence of results with the different outcomes, independence from confounding variables, and previous reports. Statistica 7.0 software (Statsoft, Tulsa, OK, USA) was used for the analyses.

Data availability: all data included in this study is available in the S1 Dataset.

## Results

### Characteristics of the RA patients

Fifteen patients lacking baseline DAS28 information were excluded. The remaining 566 RA patients showed the demographic and clinical characteristics shown in Table 1. They presented a severe disease before the start of TNFi, as indicated by the frequent presence of erosions and of the specific RA autoantibodies, and by the high mean disease activity (mean DAS28 > 5.1) that was not controlled by previous treatments. The patients were evaluated during the course of treatment with infliximab (n = 381), adalimumab (n = 152) or etanercept (n = 33). Response to treatment was evaluated at 3, 6 or 12 months of follow-up with information available for a variable number of patients at each point (n = 289 at 3 months, 532 at 6 months and 365 at 12 months). All of them except 2.6% received TNFi together with conventional antirheumatic drugs. The treatment was associated with a significant decrease in RA activity, which was more marked during the first 3 months than at later times. The mean DAS28 value remained in a narrow range from the 3^rd^ month onward (4.1 to 3.7). In a similar way, the frequency of non-responders according to the EULAR criteria was very uniform at the three assessment times (21.1 to 23.0%).

**Table 1:** Clinical characteristics of the 566 patients with RA.

Characteristic	Value
Women, N (%)	478 (84.5)
Age at treatment, mean ± SD	52.3 ± 12.8
Rheumatoid factor positive, N (%)	410 (72.6)
Anti-CCP positive, N (%)	328 (67.8)
Erosive arthritis ^a^, N (%)	284 (68.4)
Smoking ^a^, N (%)	57 (17.1)
Concomitant cDMARD ^a^, N (%)	455 (97.4)
TNFi, N (%)	
infliximab	381 (67.4)
adalimumab	152 (26.8)
etanercept	33 (5.8)
Baseline ESR ^a^, mean ± SD	38.3 ± 22.9
Baseline CRP ^a^, mean ± SD	11.9 ± 20.1
Baseline HAQ ^a^, mean ± SD	1.54 ± 0.68
DAS28, mean ± SD	
Baseline	5.7 ± 1.2
3 months	4.1 ± 1.4
6 months	3.8 ± 1.4
12 months	3.7 ± 1.5
EULAR response, N (%)	
3 months, N = 289	
Good responder	72 (25.0)
Moderate	150 (52.0)
Non responder	67 (23.0)
6 months, N = 532	
Good responder	187 (35.1)
Moderate	227 (42.7)
Non responder	118 (22.2)
12 months, N = 365	
Good responder	150 (41.1)
Moderate	138 (37.8)
Non responder	77 (21.1)

Abbreviations: N, number; SD, standard deviation; anti-CCP, anti-cyclic citrullinated peptide antibodies; cDMARDs, conventional disease-modifying anti-rheumatic drugs; HAQ, health assessment questionnaire; DAS28, Disease Activity Score 28 joints; ESR, erythrocyte sedimentation rate; CRP, C-reactive protein; EULAR, The European League Against Rheumatism.

^a^ Data from <85% of the patients were available: 415 for erosive arthritis, 334 for smoking, 467 for concomitant cDMARDs, 375 for baseline ESR, 378 for baseline CRP and 372 for baseline HAQ.

### Analysis of association with response to TNFi

Univariate analyses identified clinical and demographic variables that were associated with ΔDAS28 at 6 months (Table 2). The strongest association was found between ΔDAS28 and baseline DAS28. It was followed by the specific TNFi used, and by sex. These three variables retained association in the multivariate analysis including them (Table 2). Therefore, they were retained as potential confounders for all analyses. No association between ΔDAS28 at 6 months and age at the start of treatment, time since disease onset, presence of autoantibodies (rheumatoid factor or anti-cyclic citrullinated peptides) or erosions, smoking, concomitant antirheumatic drug, or baseline disability was observed (Table 2).

**Table 2:** Characteristics of the patients associated with ΔDAS28 at 6 months in either univariate or multivariate regression.

	Univariate		Multivariate	
Characteristic	β (SE)	p	β (SE)	p
Sex	-0.07 (0.04)	0.09	-0.09 (0.04)	0.025
Age at treatment	-0.006 (0.05)	0.9		
Time since diagnosis	-0.06 (0.05)	0.2		
Rheumatoid factor	-0.01 (0.04)	0.8		
Anti-CCP	-0.03 (0.05)	0.6		
Erosive arthritis	-0.06 (0.05)	0.3		
Smoking	-0.06 (0.06)	0.3		
Concomitant cDMARD	0.02 (0.05)	0.6		
infliximab/other	0.10 (0.04)	0.02	0.19 (0.04)	0.000002
Baseline HAQ	0.06 (0.05)	0.2		
Baseline DAS28	0.42 (0.04)	< 10^−16^	0.47 (0.04)	< 10^−16^

Abbreviations: β, coefficient of the regression; SE, standard error; anti-CCP, anti-cyclic citrullinated peptide antibodies; cDMARDs, conventional disease-modifying anti-rheumatic drugs; HAQ, health assessment questionnaire; DAS28, Disease Activity Score 28 joints.

Two of the 18 SNPs failed quality control and were excluded from analysis. None of the remaining 16 SNPs was associated with response to TNFi in our patients when the threshold of significance was adjusted for the number of tests (p < 0.003). Specifically, none of the SNPs was associated with the primary outcome, ΔDAS28 at 6 months, or with the secondary outcomes ΔDAS28 at 3 or at 12 months, even at the level of the uncorrected 0.05 p value (Table 3). Regarding the secondary outcomes based in EULAR criteria, only a SNP showed nominal association with EULAR non-response (rs10925026 at 6 months), but it was not below the adjusted p value, and the direction of changes was not consistent at other assessment times (Table 4).

**Table 3:** Results of the linear regression between the studied SNPs and ΔDAS28 at 3, 6 and 12 months of treatment ^a^.

			3 mo.			6 mo.		12 mo.	
Locus	SNP	Allele	β ^b^		p	β ^b^	p	β ^b^	p
*ALPL*	rs885813	C	- 0.04	0.5		0.01	0.8	- 0.05	0.3
	rs885814	T	- 0.03	0.5		0.0006	1.0	- 0.06	0.2
*CARD8*	rs10403848	A	- 0.08	0.13		- 0.02	0.5	- 0.03	0.6
	rs11672725	T	- 0.01	0.8		- 0.01	0.9	- 0.03	0.5
*GFRA1*	rs7070180	T	- 0.02	0.7		- 0.07	0.07	- 0.005	0.9
*LRPAP1*	rs3468	A	0.02	0.7		0.03	0.5	- 0.04	0.3
*LRRC55*	rs717117	G	- 0.01	0.8		- 0.02	0.6	0.03	0.6
*MAP2K6*	rs11870477	C	0.02	0.7		0.02	0.6	0.05	0.3
*MAP3K7*	rs284511	T	- 0.03	0.6		- 0.05	0.2	- 0.05	0.3
	rs284515	G	- 0.06	0.3		0.01	0.8	- 0.04	0.4
*NLRP3*	rs4925659	A	- 0.03	0.6		- 0.01	0.7	- 0.004	0.9
	rs4925648	T	0.03	0.5		0.05	0.2	- 0.04	0.4
	rs10925026	C	- 0.01	0.8		- 0.02	0.7	0.02	0.7
	rs4612666	T	- 0.03	0.6		0.02	0.7	- 0.005	0.9
*NR2F2*	rs16973982	C	0.06	0.3		0.07	0.08	0.07	0.11
*PDE3A-SLCO1C1*	rs3794271	G	- 0.04	0.5		- 0.01	0.7	0.05	0.3

^a^ Linear regression was adjusted by baseline DAS28, TNFi and sex.

^b^ All β standard errors were = 0.05 for analyses at 3 and 12 months, and they were = 0.04 for all analyses at 6 months.

**Table 4:** Results of the logistic regression between the studied SNPs and the non-responder EULAR class at 3, 6 and 12 months of treatment ^a^.

		3 mo.		6 mo.		12 mo.	
SNP	Allele	OR (CI 95%)	p	OR (CI 95%)	p	OR (CI 95%)	p
rs885813	C	0.9 (0.5–1.5)	0.6	1.0 (0.6–1.5)	0.8	1.1 (0.6–1.8)	0.8
rs885814	T	1.0 (0.6–1.6)	1.0	1.0 (0.7–1.4)	0.9	1.4 (1.0–2.1)	0.07
rs10403848	A	1.3 (0.7–2.1)	0.4	1.2 (0.8–1.8)	0.3	1.4 (0.8–2.3)	0.2
rs11672725	T	1.1 (0.7–1.7)	0.7	1.0 (0.7–1.5)	0.8	1.2 (0.8–1.9)	0.4
rs7070180	T	1.2 (0.7–2.1)	0.6	1.3 (0.9–2.1)	0.2	1.0 (0.6–1.6)	0.9
rs3468	A	0.9 (0.5–1.5)	0.6	0.7 (0.5–1.1)	0.12	0.9 (0.5–1.6)	0.8
rs717117	G	1.5 (0.6–3.7)	0.4	1.2 (0.6–2.3)	0.6	0.9 (0.3–1.9)	0.7
rs11870477	C	1.2 (0.7–2.1)	0.6	1.0 (0.6–1.5)	0.9	0.7 (0.4–1.3)	0.3
rs284511	T	1.3 (0.9–1.9)	0.3	1.0 (0.8–1.4)	0.9	1.2 (0.8–1.7)	0.4
rs284515	G	1.2 (0.7–2.0)	0.5	0.9 (0.6–1.4)	0.7	1.4 (0.8–2.3)	0.3
rs4925659	A	1.3 (0.9–2.0)	0.2	0.8 (0.6–1.1)	0.2	1.2 (0.8–1.8)	0.4
rs4925648	T	0.9 (0.4–1.7)	0.7	0.7 (0.4–1.1)	0.10	1.0 (0.6–1.7)	1.0
rs10925026	C	0.9 (0.6–1.4)	0.6	1.4 (1.0–1.9)	0.037	0.9 (0.6–1.3)	0.5
rs4612666	T	1.3 (0.9–2.0)	0.2	0.9 (0.6–1.3)	0.5	1.0 (0.7–1.5)	1.0
rs16973982	C	1.1 (0.5–2.0)	0.9	0.9 (0.5–1.5)	0.7	0.9 (0.5–1.6)	0.6
rs3794271	G	1.2 (0.8–1.8)	0.4	0.7 (0.5–1.0)	0.06	0.8 (0.5–1.2)	0.4

^a^ Logistic regression was adjusted for baseline DAS28, the specific TNFi and sex

Abbreviations: OR, odds ratio; CI, confidence interval

### Direct comparison with previous studies

We also checked the consistency of our results with the previous studies in which the biomarkers had been proposed [7,8,14–18]. This implied to consider the same time of assessment, measure of response, reference allele, and direction of change (improvement or lack of improvement) that produced the lowest p value in previous studies (Table 5). In addition, we considered the comparability of the minor allele frequencies (MAF), the proportions of patients on each specific TNFi and on concomitant treatment with cDMARD, and the fraction of ACPA positive patients, as all these factors could influence the association.

**Table 5:** Direct comparison of results for the same outcomes in the current and previous studies. Outcomes were either ΔDAS28 at 3 or 6 months, or the EULAR criteria, which were used as a comparison of non-responders (NR) with responders (good + moderate responders (GR + M)) at 3 months, or a comparison of NR with GR either at 3 or 6 months. Only previous studies with significant associations are included. Comparison of effect size and direction, and of p values.

		Effect size		p		
Outcome	SNP	Current	Previous	Current	Previous	Reference
ΔDAS28		β	β			
3 mo.	rs885813	- 0.04	-	0.5	1.0 x10^-5^	14
	rs885814	- 0.03	-	0.5	5.5 x10^-6^	14
	rs10403848	- 0.08	0.18	0.13	0.02	15
	rs11870477	0.02	-	0.7	3.3 x10^-6^	14
	rs284515	- 0.06	0.35	0.3	5.6 x10^-5^	8 ^a^
	rs16973982	0.06	-	0.3	2.8 x10^-6^	14
6 mo.	rs7070180	0.07 ^b^	0.30	0.07	6.4 x10^-5^	16
	rs284511	0.05 ^c^	0.41	0.2	2.5 x10^-8^	8
NR *vs*. (GR+M)		OR	OR			
3 mo.	rs11672725	0.92 ^b^	1.05	0.7	0.032	15
	rs4925659	0.77 ^b^	1.06	0.2	0.006	15
	rs10925026	1.12 ^b^	0.94	0.6	0.017	15
	rs4612666	0.75 ^b^	0.64	0.2	0.025	17
NR *vs*. GR						
3 mo.	rs717117	1.36	10.7	0.4	9.6 x10^-6^	14
	rs4925648	1.00 ^b^	1.15	1.0	0.035	15
	rs3794271	1.28	3.2	0.3	3.5 x10^-6^	14
			2.63		1.7 x10^-5^	7
6 mo.	rs3468	0.8	1.28	0.4	0.003	18

^a^ An even lower p value was reported in this study with the joint analysis of response at 3 and at 6 months.

^b^ Referred to the opposite outcome to match previous analysis.

^c^ Referred to the opposite allele, because the minor allele is different in the Japanese and the Europeans.

First, we compared the direction of effect. Only 5 of the 12 SNPs, in which this information was available from the previous studies, showed consistency in direction between our results and the previous reports, one was completely neutral (rs4925648), and the other 6 SNPs showed opposite direction (Table 5). Next, we focused in the effect sizes of the 5 SNPs showing the same direction of effect in the two studies. For the 2 SNPs assessed as ΔDAS28 (rs7070180, rs284511), the regression coefficients were markedly lower in our study than in the previous ones. Also, for the 3 SNPs assessed according with EULAR criteria (rs4612666, rs717117, rs3794271), the odds ratios were notably nearer 1.0 in our results than in previous reports. Information of direction or size of effect for the remaining 4 SNPs (rs885813, rs885814, rs11870477, rs16973982) was not available in the previous report [14], and only comparison of the p values was possible. The contrast between p values for these 4 SNPs was very notable, because none of them showed even a trend to association in our results, but showed p values ≤ 1 x10^-5^ in the previous report [14].

We also found the MAF of most of the SNPs in our patients were similar to the observed in the previous studies reporting association (Table A in S1 Text). All the MAF were less than 20% different from the previously reported in Europeans, except for rs717117 that was about half as frequent in our patients as in the Danish patients from the previous report [14]. The MAF differences were larger (+30% and -33%) for the two SNPs, rs284511 and rs284515, previously reported in Japanese patients [8]. Additionally, our patients were comparable with previous ones in the percentage receiving concomitant treatment with cDMARD, which ranged from 73 to 87% except for a study with all patients on combined treatment [16], and in the fraction of ACPA positive patients, which ranged from 59 to 89% (Table B in S1 Text). However, there was a lot of heterogeneity in the distribution of the specific TNFi (Table B in S1 Text). Five studies (three of which were from the same patient registry) showed an even distribution in three fractions receiving either infliximab, adalimumab or etanercept [7,15,17,18,24]. Two studies, including the current one, showed about 70% of patients on infliximab and 25% on adalimumab with only 5% on etanercept [14]. Other two studies were dominated by patients on infliximab or etanercept, with only 10% on adalimumab [8,16]; and finally, a unique study included 65% of patients treated with adalimumab, with the remaining patients distributed between the other two TNFi [25].

## Discussion

Our attempt to validate genetic biomarkers of response to TNFi has led to increased doubts on their status. Consequently, our results emphasize the need of independent validation of all genetic findings, even of those that reach the GWAS level of significance. In addition, the results call for a revision of the ideas justifying the search of genetic biomarkers, and to consider possible ways to improve their reproducibility.

At a first glance, the most surprising lack of association in our patients corresponds to rs3794271 in *PDE3A-SLCO1C1*, and rs284511 near *MAP3K7*, because the two SNPs have reached p < 5 x10^-8^, the GWAS threshold of significance, in previous studies [7,8]. A more detailed consideration, yet, indicates that our results are not so striking. In relation with rs3794271, because its role as biomarker has already been contradicted by lack of association with response to TNFi in 1750 UK patients with RA [24]. This study was much larger than the two studies that discovered this putative biomarker (196 Danish and 315 Spanish patients, respectively) [7,14]. A fraction of the patients in the UK study differed from the patients in the two other studies, but these differences seemed irrelevant for the lack of replication [24]. Our study, which was in all respects comparable to the Danish and Spanish studies [7,14], reinforces this conclusion. In relation to the other SNP, rs284511 (and rs284515 in the same *MAP3K7* locus), our study is the first independent validation attempt, but the possibility of an ethnic factor in the discordant results should be reckoned as it was discovered in Japanese patients [8]. An ethnic factor is well known in the genetic component of RA, where most loci are shared between Europeans and Asians, but 5 loci are specific to one or the other ethnic group [26]. This possibility was also suggested by the difference in MAF of the two *MAP3K7* SNPs between our study and the Japanese patients, which was larger than the present for SNPs from European reports. Asian specificity of the *MAP3K7* association would explain our result and that none of the previous GWAS in Europeans have mentioned these two SNPs [12–14,16,25,27].

Other 5 SNPs were initially discovered in the same Danish GWAS that uncovered the already discussed SNP in *PDE3A-SLCO1C1* [14]. All of them showed p < 10^−4^ in the 196 patients of that study, but none obtained subsequently any support. The low frequency rs717117 SNP has never been reported again. None of the other 4 SNPs was associated with response to TNFi in a GWAS of 882 RA patients from the Netherlands [25], and rs11870477 was not replicated in 315 patients of a Spanish study [7]. This latter study did not analyze the other 4 SNPs.

More intriguing is the status of rs7070180 in the *GFRA1* gene, whose investigation has been prone to mishaps. This SNP showed significant association (p < 10^−4^) with response to TNFi in stages 1 and 2 of a UK study (945 patients in total), but its assay failed in stage 3 [16]. Subsequent studies never mentioned it, until a recent GWAS in Japanese patients [8]. This study identified rs1679568, other SNP in the same *GFRA1* locus, as associated with response to TNFi with p < 10^−6^. The authors took this result as concordant with the rs7070180 association in UK patients because rs7070180 was not genotyped or imputed in the Japanese patients [8]. Regrettably, our results were also incomplete. On one side, the rs1679568 assay did not pass quality control in our study. On the other, our results for rs7070180 where inconclusive: we found a smaller effect size than in Plant *et al*. (β = 0.07 *vs*. 0.30) [16], but concordant direction of effect and a trend to association (p = 0.07). Consequently, we think the status of this locus is still ambiguous.

The remaining seven SNPs had shown association with response to TNFi that was significant according with the criteria applied to their discovery studies [15,17,18], but that was less convincing from the statistical point of view (p values between 0.05 and 0.003) than the SNPs discussed above. The additional criteria leading to take these SNPs as potential biomarkers included functional studies that established a correlation between the SNPs and molecular changes that seemed important for RA pathogenesis. Six of these SNPs are located in two interesting genes from the point of view of RA pathogenesis, *NLPR3* (four SNPs) and *CARD8* (two SNPs). Five of them were associated with response to TNFi in a study of 1278 UK patients with RA [15]. In the same study, the authors found differences in expression of the *NLPR3* and *CARD8* genes between monocytes from RA patients and controls, and association linking the SNPs with the expression of *NLPR3* and *CARD8* in monocytes [15]. Further support for association of *NLPR3* with the response to TNFi was provided by an independent study of 538 Danish patients with RA [17]. In this study, only a *NLPR3* SNP (rs4612666) was tested, but it showed association. The final SNP of the seven in this group (rs3468) was selected from a very attractive study. In it, the A allele of rs3468 was identified primarily as the SNP that was most associated (p < 1.1 x10^-6^) with increased DNA methylation at two cis-CpG sites on the *LRPAP1* gene, which, in turn, was strongly associated with response to etanercept (p < 1.7 x10^-8^). In addition, there was weak genetic association (p < 0.05) of the A allele of rs3468 with non-response to TNFi in two sets of RA patients (with 56 and 1204 patients, respectively) [18]. Therefore, the relationship was established mainly as indirect. Our results were clearly contrary to the previously reported and, together with lack of replication of the other six SNPs in this group, reinforce the well-known fact that the main determinant of reproducibility of genetic associations is the level of statistical association [28,29].

In addition to the specific comments for each SNP of the previous paragraphs, there are other factors that could have contributed to the lack of reproducibility of the results. The possible impact of differences in ethnicity has already been discussed in relation with the *MAP3K7* SNPs. All other SNPs were described in studies done with European patients and ethnicity is less likely a factor for them. In addition, MAF were comparable for all the SNPs from European studies except for rs717117, whose low frequency in our patients (MAF = 5.3%) made for a lower statistical power than in the original study (MAF = 11%). Other study characteristics that could impinge in the results, the fraction of patients on concomitant treatment with cDMARD and the fraction with ACPA, can also be dismissed as differential because they were comparable between studies. Nevertheless, there was marked variability in the distribution of the specific TNFi that the patients had received. No common pattern was found among the reports, with our patients being only comparable with a previous study. The two sets of patients showed predominant treatment with infliximab and few patients on etanercept. The other studies were characterized by a variety of TNFi distributions. Considering that some patients who are resistant to a TNFi respond to a different one, it is possible that these differences in representation of the various TNFi have contributed to the lack of replication.

Our results invite to reconsider the search of genetic biomarkers of response to TNFi. The first point to ponder is the heritability of the phenotype, which was unknown until recently, when a familial component in the response to TNFi was demonstrated [30], and the heritability of ΔDAS28 was quantified (h^2^ from 0.59 to 0.71 in different estimations) as sufficient for genetic studies [31]. However, change in the number of swollen joints or in the number of tender joints, which are components of DAS28, showed larger heritability than ΔDAS28 suggesting that they could be used as more discriminant outcomes of treatment [31]. Possible benefits of these alternative outcomes remain still untested, and we could not assess their performance in our study because they are not available in many of our patients. Other improvement that has been proposed consist in reducing within patient variability by using as outcome the averages of frequently repeated response assessments [32]. Another point of interest is the structure of the genetic component of response to TNFi, which has been clarified by recent studies [27,33,34]. This component is not significantly shared with the RA susceptibility loci, either taken individually, or in combination [9,33]. In addition, rare variants with strong effect do not contribute significantly to the response to TNFi [34]. Therefore, only common variants unrelated with RA susceptibility seem likely, but the effect of each of them appears to be small. This is the conclusion of a thorough study, with many analysis tools and by multiple teams, done in 2706 RA patients [27]. This conclusion emphasizes the need to continue searching with more GWAS, which are very powerful for identifying common genetic variants but need to be large to find those of small effect. Also, it emphasizes the importance of pursuing the best supported biomarkers to establish their value, as *PTPRC* [9–11], a recently discovered SNP associated with response to etanercept [13], the SNPs associated in the Japanese GWAS [8], or other SNPs with significant association in recent meta-analysis [5]. In addition, it is to be expected that biomarkers will be searched in other ethnicities as we lack information about many populations that could show particular biomarkers of value for the RA patients from the corresponding genetic background.

The available evidence, therefore, shows the need for prudence in claiming new biomarkers, the requirement for independent replication of all previous findings, and the convenience of exploring the possibility of drug-specific biomarkers among the TNFi, and the use of outcomes with high heritability, as change in the number of swollen or tender joints, and of using averages of repeated assessment of these outcomes, to increase the success of genetic studies. Progress along these lines will require new and large collections of patients.

## Supporting information

S1 Text Information on selected previous studies and compatibility with the current study. It includes two tables. Table A: List of SNPs selected for this study with references and the corresponding quality control results in the current study. Table B: Characteristics of the patients included in previous studies compared with the current study.
(DOCX)

S1 Dataset Raw data with all the variables and patients considered in this study. (XLSX)
