# Supplementary material for: Validation study of genetic biomarkers of response to TNF inhibitors in rheumatoid arthritis
Source: PLoS One. 2018 May 7;13(5):e0196793. doi: 10.1371/journal.pone.0196793 (PMC5937760; doi:10.1371/journal.pone.0196793)
Supplement: S1 Text — It includes two tables. Table A: List of SNPs selected for this study with references and the corresponding quality control results in the current study. Table B: Characteristics of the patients included in previous studies compared with the current study. (DOCX) [file pone.0196793.s001.docx]

**Table A.** List of SNPs selected for this study with references and the corresponding quality control results in the current study ^a^. MAF from the previous references analyzing the same SNP for response to TNFi are provided for comparison as original MAF.

| Locus | SNP | References | Allele | MAF | Original MAF | % difference | HWE | CEU | IBS |
| --- | --- | --- | --- | --- | --- | --- | --- | --- | --- |
| *ALPL* | rs885813 ^b^ | [[1](#_ENREF_1),[2](#_ENREF_2)] | T/C | 0.49 | 0.46/0.43 | 10 | 0.4 | 0.54 | 0.56 |
|  | rs885814 | [[1](#_ENREF_1),[2](#_ENREF_2)] | C/T | 0.28 | 0.32/0.32 | -16 | 0.11 | 0.27 | 0.30 |
| *CARD8* | rs10403848 | [[3](#_ENREF_3)] | G/A | 0.18 | 0.21 | -20 | 0.9 | 0.20 | 0.15 |
|  | rs11672725 | [[3](#_ENREF_3)] | C/T | 0.21 | 0.19 | 10 | 0.9 | 0.18 | 0.19 |
| *GFRA1* | rs7070180 | [[4](#_ENREF_4)] | C/T | 0.31 | 0.25 | 18 | 0.4 | 0.28 | 0.26 |
|  | rs1679568 ^c^ | [[5](#_ENREF_5)] | G/A | 0.22 | 0.15 | 32 | 0.003 | 0.17 | 0.21 |
| *LRPAP1* | rs3468 | [[6](#_ENREF_6)] | G/A | 0.37 | 0.35 ^d^ | 6 | 0.5 | 0.32 | 0.34 |
| *LRRC55* | rs717117 | [[1](#_ENREF_1)] | A/G | 0.05 | 0.11 | -108 | 1.00 | 0.06 | 0.03 |
| *MAP2K6* | rs11870477 | [[1](#_ENREF_1),[2](#_ENREF_2),[7](#_ENREF_7)] | A/C | 0.12 | 0.15/0.13/0.15 | -19 | 0.01 | 0.12 | 0.11 |
| *MAP3K7* | rs284511 | [[5](#_ENREF_5)] | C/T | 0.47 | 0.33 | 30 | 0.4 | 0.44 | 0.51 |
|  | rs284515 | [[5](#_ENREF_5)] | A/G | 0.13 | 0.17 | -33 | 0.13 | 0.11 | 0.12 |
| *NLRP3* | rs4925659 | [[3](#_ENREF_3)] | G/A | 0.40 | 0.41 | -2 | 0.8 | 0.44 | 0.34 |
|  | rs4925648 | [[3](#_ENREF_3)] | C/T | 0.12 | 0.12 | 0 | 1.00 | 0.09 | 0.11 |
|  | rs10925026 | [[3](#_ENREF_3)] | A/C | 0.33 | 0.34 | -4 | 0.3 | 0.32 | 0.33 |
|  | rs4612666 | [[3](#_ENREF_3),[8](#_ENREF_8)] | C/T | 0.31 | 0.26/0.26 | 16 | 0.4 | 0.21 | 0.27 |
| *NR2F2* | rs16973982 | [[1](#_ENREF_1),[2](#_ENREF_2)] | T/C | 0.12 | 0.11/0.14 | -9 | 0.01 | 0.09 | 0.11 |
|  | rs10520789 ^c^ | [[1](#_ENREF_1),[2](#_ENREF_2),[7](#_ENREF_7)] | G/A | 0.13 | 0.11/0.13/0.11 | 11 | 0.003 | 0.10 | 0.09 |
| *PDE3A-SLCO1C1* | rs3794271 | [[1](#_ENREF_1),[7](#_ENREF_7),[9](#_ENREF_9)] | A/G | 0.35 | 0.35/0.35/0.40 | -4 | 0.7 | 0.43 | 0.35 |

^a^ Allele: major/minor allele; MAF, minor allele frequency; HWE, Hardy-Weinberg equilibrium p value; CEU, MAF in Utah residents with Northern and Western European ancestry from HapMap; IBS, MAF in Iberian populations in Spain from HapMap.

^b^ The C allele was the minor allele in our study (0.495); however, in CEU and IBS it was the major allele (0.545 and 0.556, respectively).

^c^ These SNPs were excluded due to lack of concordance with HWE.

^d^ MAF for this SNP was not available in reference [6], this MAF was obtained from the EU samples of the 1000 Genomes Project.

**Table B.** Characteristics of the patients included in previous studies compared with the current study.

| Study | Country ^a^ | Infliximab | Adalimumab | Etanercept | Other TNFi ^b^ | Combined ^c^ | ACPA^+^ |
| --- | --- | --- | --- | --- | --- | --- | --- |
| Current study | ES+GR | 67% | 27% | 6% | 0% | 80% | 68% |
| [1] | DK | 72% | 21% | 6% | 0% | 87% | 59% |
| [2] | NL | 23% | 65% | 12% | 0% | 73% | - |
| [3] ^d^ | UK | 39% | 40% | 21% | 0% | - | - |
| [4] | UK | 43% | 14% | 43% | 0% | 100% | - |
| [5] | JP | 55% | 7% | 39% | 0% | 80% | 89% |
| [6] ^d^ | UK | 32% | 31% | 34% | 0% | - | - |
| [7] | ES | 37% | 28% | 36% | 0% | - | 79% |
| [8] | DK | 31% | 25% | 31% | 13% | 84% | 76% |
| [9] ^d^ | UK | 26% | 31% | 36% | 6% | 81% | - |

^a^ Country two-letter codes: ES = Spain, GR = Greece, DK = Denmark, NL = Netherlands, UK = United Kingdom, JP = Japan.

^b^ Other TNFi were Certolizumab pegol and Golimumab.

^c^ Concomitant treatment with cDMARD.

^{Krintel, 2012 #588}^ These three studies shared a large percentage of the same patients.

**References:**

1. Krintel SB, Palermo G, Johansen JS, Germer S, Essioux L, Benayed R, et al. Investigation of single nucleotide polymorphisms and biological pathways associated with response to TNFalpha inhibitors in patients with rheumatoid arthritis. Pharmacogenet Genomics. 2012; 22: 577-589.

2. Umicevic Mirkov M, Cui J, Vermeulen SH, Stahl EA, Toonen EJ, Makkinje RR, et al. Genome-wide association analysis of anti-TNF drug response in patients with rheumatoid arthritis. Ann Rheum Dis. 2013; 72: 1375-1381.

3. Mathews RJ, Robinson JI, Battellino M, Wong C, Taylor JC, Biologics in Rheumatoid Arthritis G, et al. Evidence of NLRP3-inflammasome activation in rheumatoid arthritis (RA); genetic variants within the NLRP3-inflammasome complex in relation to susceptibility to RA and response to anti-TNF treatment. Ann Rheum Dis. 2014; 73: 1202-1210.

4. Plant D, Bowes J, Potter C, Hyrich KL, Morgan AW, Wilson AG, et al. Genome-wide association study of genetic predictors of anti-tumor necrosis factor treatment efficacy in rheumatoid arthritis identifies associations with polymorphisms at seven loci. Arthritis Rheum. 2011; 63: 645-653.

5. Honne K, Hallgrimsdottir I, Wu C, Sebro R, Jewell NP, Sakurai T, et al. A longitudinal genome-wide association study of anti-tumor necrosis factor response among Japanese patients with rheumatoid arthritis. Arthritis Res Ther. 2016; 18: 12.

6. Plant D, Webster A, Nair N, Oliver J, Smith SL, Eyre S, et al. Differential Methylation as a Biomarker of Response to Etanercept in Patients With Rheumatoid Arthritis. Arthritis Rheumatol. 2016; 68: 1353-1360.

7. Acosta-Colman I, Palau N, Tornero J, Fernandez-Nebro A, Blanco F, Gonzalez-Alvaro I, et al. GWAS replication study confirms the association of PDE3A-SLCO1C1 with anti-TNF therapy response in rheumatoid arthritis. Pharmacogenomics. 2013; 14: 727-734.

8. Sode J, Vogel U, Bank S, Andersen PS, Hetland ML, Locht H, et al. Genetic Variations in Pattern Recognition Receptor Loci Are Associated with Anti-TNF Response in Patients with Rheumatoid Arthritis. PLoS One. 2015; 10: e0139781.

9. Smith SL, Plant D, Lee XH, Massey J, Hyrich K, Morgan AW, et al. Previously reported PDE3A-SLCO1C1 genetic variant does not correlate with anti-TNF response in a large UK rheumatoid arthritis cohort. Pharmacogenomics. 2016; 17: 715-720.
